# Supplementary material for: The causal relationship between the human gut microbiota and pyogenic arthritis: a Mendelian randomization study
Source: Front Cell Infect Microbiol. 2024 Nov 26;14:1452480. doi: 10.3389/fcimb.2024.1452480 (PMC11629706; doi:10.3389/fcimb.2024.1452480)
Supplement: Supplementary file 1 [file DataSheet1.zip › mendelian test/ebi-a-GCST90027503.csv_forest.pdf]

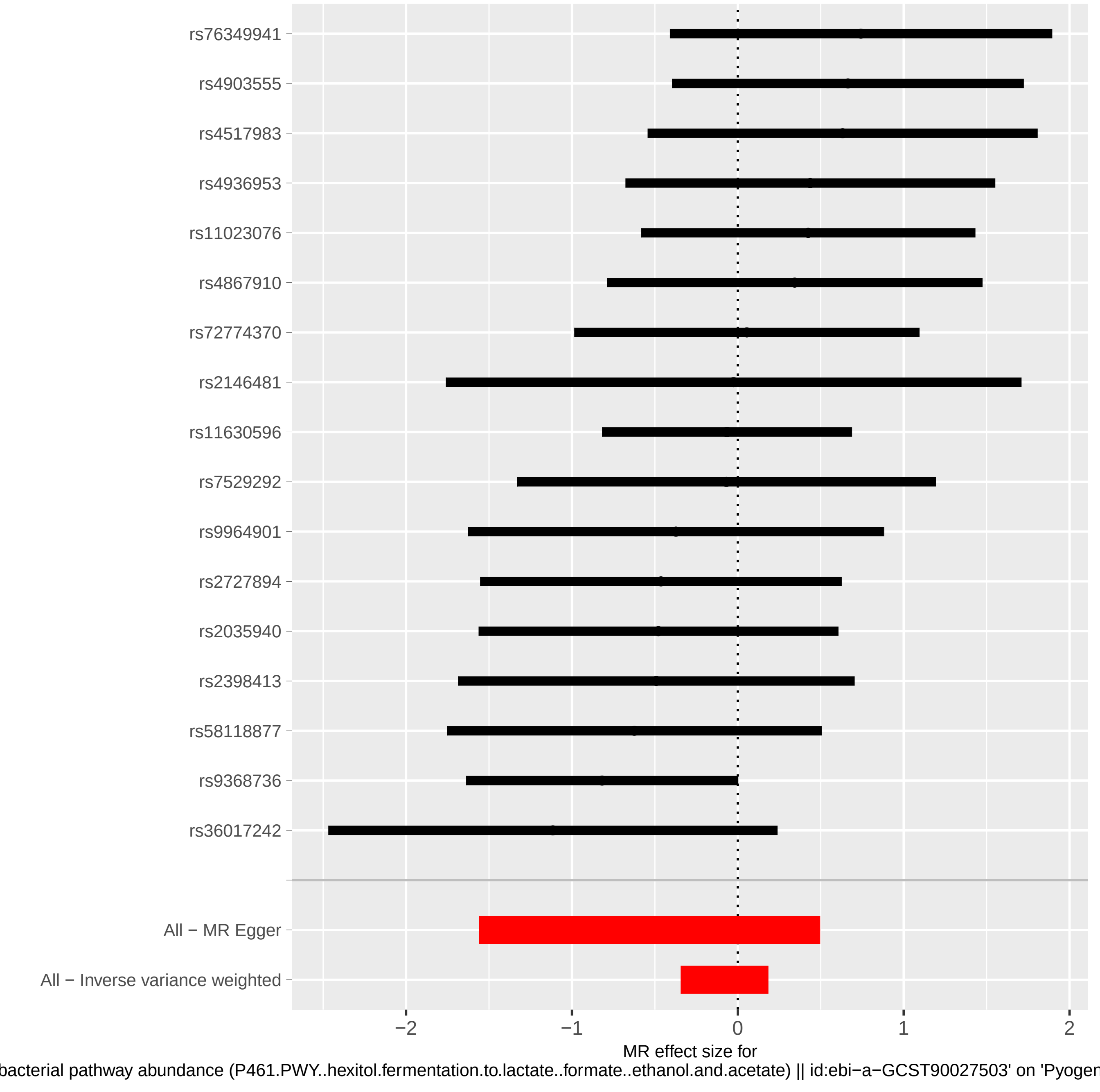
rs76349941
rs4903555
rs4517983
rs4936953
rs11023076
rs4867910
rs72774370
rs2146481
rs11630596
rs7529292
rs9964901
rs2727894
rs2035940
rs2398413
rs58118877
rs9368736
rs36017242
All − MR Egger
All − Inverse variance weighted
−2
−1
0
1
2
MR effect size for
bacterial pathway abundance (P461.PWY..hexitol.fermentation.to.lactate..formate..ethanol.and.acetate) || id:ebi−a−GCST90027503' on 'Pyogen
